# Plasma Disappearance Rate of Indocyanine Green for Determination of Liver Function in Three Different Models of Shock

**DOI:** 10.3390/diagnostics9030108

**Published:** 2019-08-31

**Authors:** Alexander Mathes, Christopher Plata, Hauke Rensing, Sascha Kreuer, Tobias Fink, Alexander Raddatz

**Affiliations:** 1 Department of Anesthesiology and Intensive Care Medicine, University Hospital Cologne, Kerpener Str. 62, 50937 Cologne, Germany; 2 Department of Anesthesiology and Intensive Care Medicine, Leopoldina Hospital Schweinfurt, Gustav-Adolf-Str. 8, 97422 Schweinfurt, Germany; 3 Department of Anesthesiology, Critical Care and Pain Medicine, Saarland University Hospital, 66424 Homburg (Saar), Germany

**Keywords:** liver function, PDR_ICG_, endotoxemia, hemorrhagic shock

## Abstract

The measurement of the liver function via the plasma disappearance rate of indocyanine green (PDR_ICG_) is a sensitive bed-side tool in critical care. Yet, recent evidence has questioned the value of this method for hyperdynamic conditions. To evaluate this technique in different hemodynamic settings, we analyzed the PDR_ICG_ and corresponding pharmacokinetic models after endotoxemia or hemorrhagic shock in rats. Male anesthetized Sprague-Dawley rats underwent hemorrhage (mean arterial pressure 35 ± 5 mmHg, 90 min) and 2 h of reperfusion, or lipopolysaccharide (LPS) induced moderate or severe (1.0 vs. 10 mg/kg) endotoxemia for 6 h (each *n* = 6). Afterwards, PDR_ICG_ was measured, and pharmacokinetic models were analyzed using nonlinear mixed effects modeling (NONMEM^®^). Hemorrhagic shock resulted in a significant decrease of PDR_ICG_, compared with sham controls, and a corresponding attenuation of the calculated ICG clearance in 1- and 2-compartment models, with the same log-likelihood. The induction of severe, but not moderate endotoxemia, led to a significant reduction of PDR_ICG_. The calculated ICG blood clearance was reduced in 1-compartment models for both septic conditions. 2-compartment models performed with a significantly better log likelihood, and the calculated clearance of ICG did not correspond well with PDR_ICG_ in both LPS groups. 3-compartment models did not improve the log likelihood in any experiment. These results demonstrate that PDR_ICG_ correlates well with ICG clearance in 1- and 2-compartment models after hemorrhage. In endotoxemia, best described by a 2-compartment model, PDR_ICG_ may not truly reflect the ICG clearance.

## 1. Introduction

Liver function may be assessed using a variety of static or dynamic tests, but the measurement of the plasma disappearance rate of indocyanine green (PDR_ICG_) has been recommended to be one of the most reliable dynamic techniques [1,2]. In critical care patients, the evaluation of PDR_ICG_ allows for a quick and easy non-invasive bed-side estimation of the liver function [3].

PDR_ICG_ has been shown to correlate well with ICG blood clearance in the critically ill [3]. ICG elimination may be a good prognostic parameter in patients after liver resection [4,5], and liver transplant dysfunction as well as mortality may be predicted by PDR_ICG_ values [6,7]. Independent of the underlying pathologies, PDR_ICG_ values of less than 8%/min are associated with a high mortality, and PDR_ICG_ values seem to correlate well with survival in critically ill patients [8,9,10].

PDR_ICG_ depends on the hepatic blood flow [11], hepatocellular uptake, and hepatocellular function. Thus, values of PDR_ICG_ may be attenuated by a reduction of the hepatic perfusion. The short-term and medium-term prediction of prognosis in patients with decompensated liver cirrhosis can be reliably performed with PDR_ICG_ [12], although the model for end stage liver disease (MELD) may perform better in specific patients [13]. However, in a recent animal study on septic shock, PDR_ICG_ failed to accurately reflect the biliary ICG excretion [14]. As a result, the authors questioned the sensitivity of PDR_ICG_ for hyperdynamic conditions. Furthermore, it was demonstrated that intrahepatic shunting may result in incorrect measurements of PDR_ICG_ [15].

PDR_ICG_ is usually calculated using data obtained from healthy volunteers, and 2-compartment models may be superior to 1-compartment models [16]. However, only limited data is available for pathologic conditions like hemorrhagic and septic shock. To fill this gap, we analyzed PDR_ICG_ and underlying compartment models after hemorrhage and resuscitation, as well as after moderate and severe endotoxemia in rats, to allow insight into ICG pharmacokinetics under these pathophysiological conditions.

## 2. Materials and Methods

### 2.1. Animals

All experiments were carried out after approval of the responsible animal use committee (permission no. LIII/180-07/2/96 and 16/2007) and in accordance with the German Animal Welfare Act. Male Sprague-Dawley rats (200–250 g body weight) were obtained from Charles River (Sulzfeld, Germany). The animals had free access to water, but pellet food was withheld for 12 h prior to surgery.

### 2.2. Surgical Procedures

Surgical procedures were carried out as described previously [17]. In short, animals were anesthetized (sodium pentobarbital 50 mg/kg intraperitoneally); an open tracheotomy was performed to facilitate spontaneous breathing. One fluid-filled PE catheter was placed in the right external jugular vein to allow for infusions and injections as described below. A second PE catheter was inserted in the left carotid artery and connected to a pressure transducer (PMSET 1DT, Becton Dickinson, Franklin Lakes, NJ, USA) for the continuous measurement of the mean arterial pressure and heart rate (Monitor Modul 66S, Hewlett Packard, Palo Alto, CA, USA). A repetitive blood gas analysis was performed using 0.2 mL arterial blood at baseline, after 90 min and at the end of the experiment (pHOx plus L; Nova Biomedical, Rodermark, Germany).

### 2.3. Experimental Protocol

**Figure 1:**
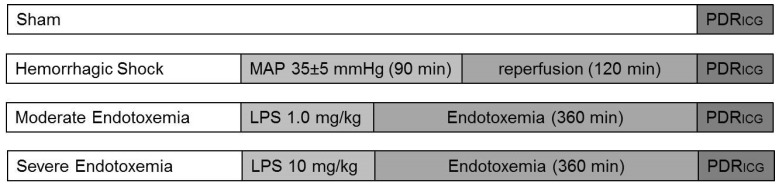
Experimental protocol. The plasma disappearance rate of indocyanine green (PDR_ICG_) was assessed in all groups, either after the sham operation, after the hemorrhagic shock, or after moderate or severe endotoxemia. LPS = lipopolysaccharide.

Figure 1 gives an overview of the experimental protocol. The sham-operated animals were infused with Ringer’s solution (10 mL/kg/h) but did not undergo hemorrhage or endotoxemia (*n* = 6). Hemorrhagic shock was induced by rapid arterial blood withdrawal by way of the carotid artery (mean arterial pressure, MAP: 35 ± 5 mm· Hg for 90 min; *n* = 6). The animals were resuscitated with 60% of shed blood, infused during the first 5 min of resuscitation, followed by 2 h of reperfusion (200% of the shed blood volume as acetated Ringer’s solution in the first hour, 100% in the second hour of reperfusion). Endotoxemia was induced by the administration of lipopolysacharide (*Escherichia coli*; O26:B6, Sigma Aldrich, Taufkirchen, Germany) at either 1.0 or 10 mg/kg intraperitoneally (each *n* = 6). After the reperfusion, or 6 h after the induction of endotoxemia, the animals underwent the measurement of the PDR_ICG_.

### 2.4. Evaluation of PDR_ICG_

The PDR_ICG_ was measured after a continuous infusion of ICG (Pulsion, Munich, Germany) at 2.5 mg/h via the jugular vein (V. jugularis externa dextra) for 60 min to achieve a steady state [18]. The amount of Ringer’s solution during this time was reduced accordingly, to compensate for the additional fluid infusion by ICG. Animals were anticoagulated with heparin (300 IU/kg) 15 min before blood sampling. Blood samples (0.3 mL) were taken at 0, 2, 4, 6, 8, 10, 15 and 20 min after stopping the infusion of ICG, covered with tinfoil to avoid photodegradation, and centrifuged at 10,000× *g* for 5 min. The ICG absorbance was determined spectrophotometrically at 800 nm. The corresponding plasma concentration was calculated using a dose-response relationship. The PDR_ICG_ is expressed as percentage decrease per minute (%/min).

### 2.5. Calculation of ICG Blood Clearance and Compartment Models

Using the program NONMEM^®^ V (GloboMax LLC, Hanover, MD, USA), the blood clearance and compartment models were modeled based on the plasma ICG absorption. Both parameters were included in a calculation as a population fit by minimizing the log likelihood, which maximizes the likelihood between the measured and the predicted parameters. For each investigated group, a calculation for 1-, 2- and 3-compartment models was performed. The models are viewed in three semi-interchangeable domains: volumes and clearances, the volume of the central compartment and micro-rate constants, and coefficients and exponents. In the 2-compartment model, the micro-rate constants are calculated as k_10_ = CL_1_/V_1_, k_12_ = CL_1_/V_2_ and k_21_ = CL_2_/V_2_.

### 2.6. Statistical Analysis

To test the statistical significance between the NONMEM^®^ population fit, the log likelihood ratio test was used [19]. The difference between log likelihood values follows a chi squared distribution. Two parameters were added for the 2-compartment model and 4 parameters for the 3-compartment model. With a probability of 0.05 and 1 degree of freedom, the value of the chi squared distribution is 5.99 for 2 parameters and 9.49 for 4 parameters. If the difference in the NONMEM^®^ objective functions for the different compartment models exceeds these values, the parameters are significant at *p* < 0.05.

Differences between groups were calculated using a one-way analysis of variance, after passing a normality test, followed by a Student-Newman-Keuls test; *p* < 0.05 was considered significant. Data are expressed as means ± standard deviation (SD). A statistical evaluation was performed using SigmaPlot^®^ (Systat Software, Erkrath, Germany).

## 3. Results

### 3.1. Hemodynamics and Blood Gas Analysis

All groups presented with comparable baseline values for MAP, as well as for the respiratory parameters, acid base state and hemoglobin content (Table 1). The decrease and recovery of MAP and hemoglobin values was significant in hemorrhagic shock animals. Hemorrhage was reversible as reflected by the recovery of MAP, heart rate, lactate levels and base excess. Endotoxemia resulted in an increase in lactate levels, and a decrease in MAP, pH and base excess in both LPS-treated groups; severe endotoxemia resulted in more significant impairments, compared with moderate endotoxemia.

### 3.2. Plasma Disappearance Rate of Indocyanine Green

**Figure 2:**
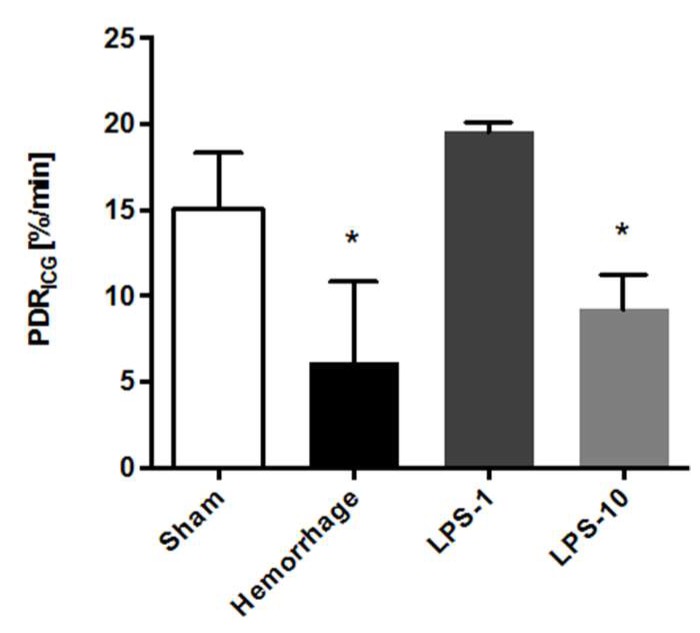
The plasma disappearance rate of indocyanine green (PDR_ICG_) was significantly reduced after hemorrhagic shock and severe endotoxemia (LPS-10), compared with the sham-operated controls and moderate endotoxemia (LPS-1). An asterisk (*) indicates *p* < 0.05 vs. sham and vs. moderate endotoxemia. LPS = lipopolysaccharide.

The sham-operated animals presented with normal PDR_ICG_ (Figure 2). The induction of hemorrhagic shock resulted in a significant attenuation of the PDR_ICG_ (*p* < 0.01 vs. sham). While moderate endotoxemia did not alter the PDR_ICG_ significantly, the administration of LPS at 10 mg/kg resulted in a PDR_ICG_ that was significantly reduced, compared with the sham operated controls (*p* < 0.01).

### 3.3. Calculation of 1-Compartment Models

The NONMEM^®^ calculation of 1-compartment models showed a reduction of the calculated ICG clearance and distribution volumes for all shock conditions, compared with the sham operated animals (Table 2). While moderate endotoxemia resulted in a moderate attenuation, hemorrhagic shock and severe endotoxemia led to an intense reduction of the calculated ICG clearance. The distribution volumes were similar for hemorrhagic shock and severe endotoxemia, while moderate endotoxemia resulted in the greatest attenuation of the distribution volume for ICG.

### 3.4. Calculation of 2-Compartment Models

**Figure 3:**
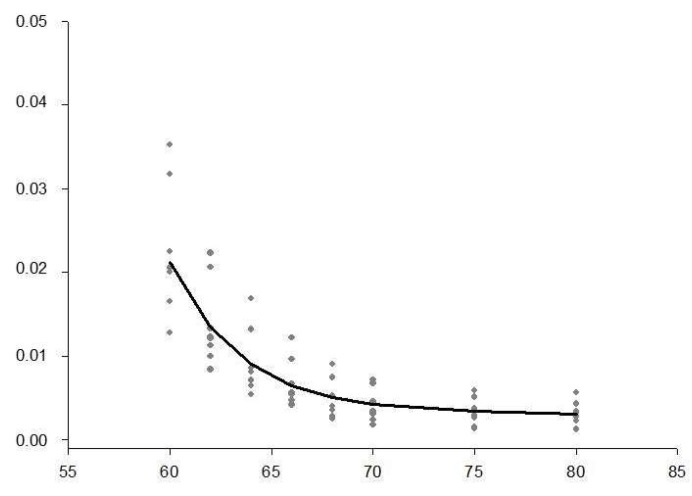
Best fit 2-compartment model for the plasma disappearance rate of indocyanine green in sham-operated animals as an example of how calculations were performed. This model allows for the estimation of the liver function in rats, but needs to be viewed with caution in hyperdynamic states.

The NONMEM^®^ calculations of 2-compartment models showed a significantly better log-likelihood for the sham-operated animals (for an example, see Figure 3), as well as for moderate and severe endotoxemia, than the 1-compartment models (*p* < 0.001) did, but not in animals that underwent hemorrhagic shock. In the 2-compartment model, the calculated ICG clearance from the first compartment was reduced after hemorrhagic shock and treatment with LPS at 10 mg/kg, while it was increased after moderate endotoxemia, compared with the sham controls (Table 2). The distribution volume of the first compartment (V1) showed similar values for the sham operated animals and both LPS conditions, whereas it displayed an increase after hemorrhagic shock. The distribution volume for the second compartment (V2) was similarly reduced after hemorrhagic shock and after moderate endotoxemia, while it was greatly attenuated after severe sepsis. The micro-rate constant k_21_ was comparable in all groups, with a slight increase in severe endotoxemia.

### 3.5. Calculation of 3-Compartment Models

In all groups, the calculation of a three-compartment model did not improve the log-likelihood over the corresponding 2-compartment models (each *p* > 0.05) (Table 2).

## 4. Discussion

In the present study, we were able to show that both hemorrhage and severe endotoxemia result in a significant reduction of the PDR_ICG_, while moderate sepsis displays values similar to sham-operated animals. The pharmacokinetic profile of ICG is best described by a two-compartment model for sham-controls, as well as for both septic conditions. After hemorrhagic shock, a one-compartment model seems sufficient to reflect the underlying PDR_ICG_ values. However, the calculated ICG clearance and PDR_ICG_ fail to correspond in a two-compartment model after septic shock.

The liver function measured by PDR_ICG_ usually shows significant attenuations following liver injury. The results obtained in this study are in good agreement with the ones reported earlier for animal models of hemorrhagic and septic shock [17,18]. However, in a porcine model, Stehr et al. demonstrated a lack of PDR_ICG_ attenuation after septic shock, thus raising the question of whether this method for the determination of the liver function may be valid under hyperdynamic conditions [14].

ICG pharmacokinetic models have been investigated before, usually concluding that two-compartment models are needed to reflect physiological ICG distribution kinetics [16]. However, in the critically ill, compartment models may be markedly altered. Our data show that for sham-operated animals and after LPS administration, the analysis of two-compartment models performed with a significant better log-likelihood compared to one-compartment models. The interpretation of three compartments did not improve the representation of the ICG distribution.

In two-compartment models, the first distribution volume (V1) most likely represents the blood volume of animals. In all experiments, V1 was within the physiological range of 70–80 mL/kg body weight for the blood volume of rats [20]. The small variations in V1 seen after hemorrhagic shock or LPS administration may well be explained by fluid shifts and volume substitution.

The second distribution volume (V2) is a virtual volume, corresponding to the cumulative extravascular volume of the ICG distribution, while clearance to this volume is referred to as C1. As we were able to show, C1 did not correlate well with PDR_ICG_ in the two-compartment model after moderate and severe endotoxemia. After 1 mg/kg of LPS, C1 was increased, while it was only gradually decreased after severe endotoxemia. These values were reflected by concomitant changes in V2: while both hemorrhagic shock and moderate sepsis led to an attenuation of V2 to a third of that of sham-controls, severe sepsis reduced the second distribution volume to less than 10 percent of the value seen in sham-operated animals.

Only in the case of hemorrhagic shock, both one- and two-compartment models were acceptable for explaining the calculated ICG clearances. Here, we also observed associated changes in the micro-rate constants that reflect liver failure after hemorrhage: the constant k_12_, indicating hepatocellular uptake, was reduced, while k_10_, corresponding to the total clearance, was greatly attenuated; both are therefore reflecting the calculated clearance and PDR_ICG_ in a similar fashion. In models of endotoxemia, these parameters did not mirror either parameter.

Interestingly, although two compartments describe the physiological [16] as well as the pathophysiological ICG distribution better than the one-compartment model for septic conditions (this study), we do not see a correlation between the PDR_ICG_ and calculated ICG clearance in animals that underwent hemorrhage. Here, the one-compartment model performs significantly better. Considering that ICG clearance mainly relies on hepatocellular uptake and hepatic perfusion, this observation certainly does not allow for the conclusion that a one-compartment model is sufficient for PDR_ICG_ calculations.

Sepsis leads to a state of hyperdynamic circulatory failure with associated alterations of the microvessel reactivity, organ perfusion and organ function. Hence, hepatic perfusion may range between hyperperfusion and sinusoidal shut-down, depending on the state and severity of the septic shock [17,18]. The hepatocellular uptake of ICG may be altered depending on the state of hepatocellular oxygenation and perfusion. Thus, it seems likely that the broad changes in liver perfusion in sepsis may alter the PDR_ICG_ results in a much more intense way than seen after hemorrhagic shock. This is supported by the finding that even changes in hepatic perfusion induced by epidural anesthesia may alter the PDR_ICG_ [11]. Furthermore, chronic conditions like liver cirrhosis may influence hepatic perfusion and consequentially PDR_ICG_ in a similar fashion [12,13]. Recent evidence suggests that liver cirrhosis may influence the PDR_ICG_ through limitations regarding hepatic perfusion and not necessarily the hepatocellular function [12]. This is in line with a recent publication showing that paracentesis in patients with liver cirrhosis may affect PDR_ICG_ measurements [21]. Thus, changes in the hepatic perfusion irrespective of the causes, like cirrhosis or sepsis, – may influence the PDR_ICG_ significantly. As a limiting condition, it has to be mentioned that the induction of an endotoxemia with LPS in our study does not strictly correlate with a microbacterial infection resulting in sepsis.

With respect to the results of this investigation, it may be argued that clinical studies have already demonstrated a good correlation between the PDR_ICG_ and ICG clearance [8,9,10]. However, it needs to be acknowledged that in all of these studies, a great variety of underlying critical conditions was included, and the number of patients with septic shock is unknown. Furthermore, although a general correlation was acceptable, the individual values of PDR_ICG_ showed a broad range of corresponding ICG blood clearances [3]. This indicates that the measurement of the liver function by means of the PDR_ICG_ may not always accurately reflect the underlying ICG blood clearances. Regarding the data presented here, this may be especially true for hyperdynamic conditions. Thus, the determination of the liver function in septic shock needs to be reviewed cautiously.

We would like to conclude that the pharmacological profile of ICG is best described by a two-compartment model after hemorrhagic and septic shock. Under septic conditions, the measurement of the liver function by PDR_ICG_ may not necessarily correspond with the ICG clearance, irrespective of the pharmacological model used for the calculation.

## Figures and Tables

**Table 1:** Blood-gas-analysis.

	Baseline	90 Min	End of Experiment
	pH		
Sham	7.37 ± 0.04	7.36 ± 0.05	7.36 ± 0.08
Hemorrhage	7.37 ± 0.04	**7.25 ± 0.07**	7.34 ± 0.06
Moderate Endotoxemia	7.36 ± 0.05	7.35 ± 0.08	**7.25 ± 0.07**
Severe Endotoxemia	7.37 ± 0.05	7.33 ± 0.09	**7.19 ± 0.09**
	Hb [g/dl]		
Sham	10.7 ± 1.1	11.0 ± 1.2	10.4 ± 1.4
Hemorrhage	11.2 ± 1.0	**6.2 ± 1.5**	8.8 ± 1.3
Moderate Endotoxemia	10.8 ± 0.8	10.0 ± 1.6	9.8 ± 1.5
Severe Endotoxemia	10.6 ± 1.2	10.1 ± 1.2	9.9 ± 1.5
	BE [mmol/L]		
Sham	0.1 ± 1.9	−0.8 ± 3.2	−0.6 ± 4.0
Hemorrhage	−0.2 ± 2.2	**−10.2 ± 3.9**	−2.0 ± 3.5
Moderate Endotoxemia	0.3 ± 1.4	−2.2 ± 1.9	**−9.7 ± 3.8**
Severe Endotoxemia	0.4 ± 1.7	−3.0 ± 2.1	**−14.2 ± 5.5**
	Lactate [mmol/L]		
Sham	1.5 ± 0.5	1.5 ± 0.4	1.6 ± 0.6
Hemorrhage	1.7 ± 0.7	**7.9 ± 1.5**	1.8 ± 0.6
Moderate Endotoxemia	1.6 ± 0.6	2.4 ± 1.2	**8.3 ± 0.7**
Severe Endotoxemia	1.7 ± 0.7	2.2 ± 1.3	**9.7 ± 0.6**

The blood gas analysis shows significant changes for different parameters at different stages of the experiment (bold numbers: *p* < 0.05 vs. corresponding baseline). Hb = hemoglobin; BE = base excess.

**Table 2:** Calculation of compartment models in different shock conditions.

	Sham	Hemorrhage	Moderate Endotoxemia	Severe Endotoxemia
	1-compartment model			
ICG Distribution Volume (mL)	60.3	41.6	32.6	42.8
Calculated ICG Clearance (mL/min/m^2^)	564.0	174.0	387.0	202.0
	2-compartment model			
ICG Distribution Volume 1 (mL)	16.2	20.2	13.3	14.1
Calculated ICG Clearance 1 (mL/min/m^2^)	246.0	84.0	305.0	181.0
ICG Distribution Volume 2 (mL)	289.9	85.6	93.3	25.5
Calculated ICG Clearance 2 (mL/min/m^2^)	211.0	170.0	90.0	467.0
Micro-rate constant k_10_	0.15	0.04	0.22	0.12
Micro-rate constant k_12_	0.13	0.08	0.06	0.33
Micro-rate constant k_21_	0.007	0.01	0.009	0.18
	3-compartment model			
ICG Distribution Volume 1 [mL]	11.7	0.01	12.8	14.1
Calculated ICG Clearance 1 [mL/min/m^2^]	137.0	134.0	300.0	181.0
ICG Distribution Volume 2 [mL]	4.37	3.85	7.89	10.00
Calculated ICG Clearance 2 [mL/min/m^2^]	1132.0	94.0	421.0	182.0
ICG Distribution Volume 2 [mL]	736.9	21.4	3.4	15.5
Calculated ICG Clearance 2 [mL/min/m^2^]	313.0	222.0	677.0	284.0

After the sham operation, moderate and severe endotoxemia, but not after hemorrhagic shock, 2-compartment models performed with a significantly better log-likelihood, compared with 1-compartment models. 3-compartment models did not perform with a significantly better log-likelihood for any intervention. ICG = indocyanine green; LPS = lipopolysaccharide.
